# Preparation of carbon black/silicone rubber composites with large-area-homogeneous-low electrical-resistance used as electroplating matrix†

**DOI:** 10.1039/d2ra06510j

**Published:** 2022-11-11

**Authors:** Yanli Dou, Shixiang Sun, Shanshan Lu, Weiguo Yao, Dongbo Guan

**Affiliations:** The Ministry of Education Key Laboratory of Automotive Material, College of Material Science and Engineering, Jilin University Changchun 130025 PR China guandb@jlu.edu.cn; China FAW Group Corporation R and D Center, First Automobile Works Group Corporation Research and Development Center Changchun 130000 PR China

## Abstract

Conductive carbon black (CCB) is an important filler in stretchable conductive silicone rubber (CSR) composites. However, due to the active oxygen-containing groups on CCB, introducing it into silicone rubber (SR) may cause SR to not completely cure. Surface modification of CCB may ease the problem but at the cost of reducing the electrical conductivity of pristine CCB. In this work, the curing and crosslinking performance of CCB/SR is detected in detail, the hydroxyl groups (–OH) carried by CCB can react with the silicon–hydrogen group (Si–H) with the existence of Pt catalyst, causing insufficiency of the hydrosilylation reaction thus hindering the solidifying process of silicon rubber. To take advantage of this reaction, more hydrogen silicone oil (PHMS) possessing silicon–hydrogen bonds is introduced into the system to improve the curing degree as well as fix the CCB in the crosslinked network. Due to the lock-in effect of CCB, the resistance of CSR samples is stable after several hundred bending cycles, and the composite's tensile strength is three times that of the pure SR samples. Besides, the size of the composites can expand to dozens of centimeters or even a few meters with uniform electric conductivity. This composite has resistance as low as 10.20 Ω and is suitable to make electroplating mold, and a rapid plating rate of 2.4 mm/24 h can be achieved. Meanwhile, the overall properties make this CSR composite have potential applications in mold manufacture, flexible electronics, and other related fields.

## Abstract 2

Carbon black/silicone rubber composites with large-area-homogeneous-low electrical-resistance used as electroplating matrix and its fine electro-deposited Ni layer.

## Graphical abstract

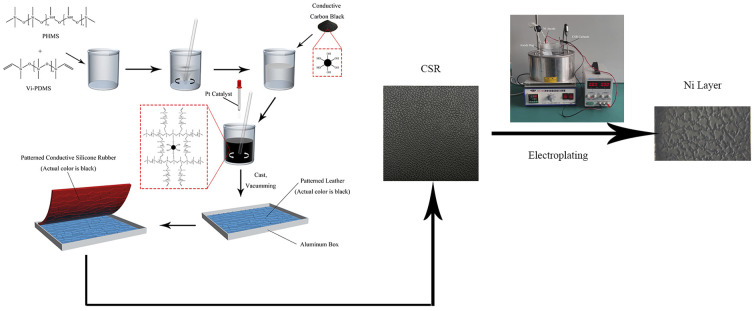

## 1. Introduction

As an indispensable component in industrial manufacture, electronics, and other areas, conductive elastomers (CEs) have fascinated researchers for so long. Different types of CEs have been developed to meet various applications. Some are used in sensor manufacturing, thus electrical sensitivity and robustness are their focus points.^1–6^ Some are made for electromagnetic interference shielding, so the promoting electromagnetic interference shielding effect is their priority;^7,8^ other applications include triboelectric nanogenerator fabrication^9–11^ and electronic skin preparation.^12–14^ Among all these researches, silicone rubber (SR) is the most frequently used matrix material. This elastomer possesses outstanding properties such as remarkable flexibility, high resistance to temperature change long service life, *etc.* Besides, some types of SR have very low viscosity and small linear shrinkage, enabling them to flow throughout the mold and automatically level itself or to fill the mold of complex shape. Thus, SR can be utilized in mold production or mold reprint in the field of archeology, medicine, and industrial production such as automotive interior manufacturing. In the automotive interior manufacturing industry, SR usually works as a medium to transfer the shape and surface details to the final metal molds. However, the traditional manufacturing process involves too many steps, and each step can cause shape or detail loss. The loss in one single process may seem bearable, but the cumulative effect can have a devastating result for the product. More importantly, the final metal mold is made of the epoxy mold with a metalized surface, and the quality of the metal mold is highly dependent on the uniformity of the surface metallization of the epoxy mold. Based on these facts, simplifying the progress is an urgent need and the easiest way to do so is to make the SR mold conductive so it can be directly electroplated. In other words, a conductive silicone rubber (CSR) that can be used for precision mold manufacturing is to be developed.

Integrating conductive fillers into the SR matrix has hitherto been the most widely used method to fabricate CSR,^15–18^ yet there are so many types of substrates and fillers to choose from. As mentioned above, to prepare the CSR we need, the SR substrate must meet the characteristics of good fluidity and small linear shrinkage after curing. These requirements bring us to the addition-cured room temperature vulcanized silicone rubber (ARTV), it has the advantages of small dimensional shrinkage and good dimensional stability compared with other types of silicone rubber. Plus, ARTV does not produce small molecules during vulcanization and has low linear shrinkage, so it is given the properties of fantastic emulation and demolding behavior, short forming cycle as well as low cost. Moreover, the characteristics of ARTV enable easier research on curing behavior for the fact that no extra substance is to be produced during its solidifying process.

Carbon conductive fillers including conductive carbon black (CCB),^19^ graphene oxide (GO),^17^ carbon nanotubes (CNTs)^18,20^ and carbon fibers (CFs)^21,22^ have been extensively used in CSR composites. Their electrical conductivities may not be comparable to their metal counterparts such as silver^7,23,24^ and copper,^25,26^ but they are relatively cheaper and have a better interaction between the filler and SR substrate.^3^ Some methods have been developed to elevate the conductivity of carbon fillers, and the most common one is surface modification such as surface coating and grafting metal.^27–29^ The benefits of surface modification are remarkable, yet new problems emerge. Some of the modified particles deteriorate in their thermal stability and some others have dispersion issues in the SR matrix.^30^ Even worse, some modified particles migrate through the substrate as time passes, causing the composites to be unstable and have short shelf lives. Apart from the pros and cons mentioned before, surface modification is inefficient, the procedure is complicated and the production rate is quite low. These disadvantages determine that surface-modified particles cannot be mass-produced, so the composite samples (no matter what matrix) are often small in size and exorbitant in cost.^3,31,32^

Herein, we present a facile preparation method of conductive silicone rubber based on an ARTV silicone oil system, and CCB was used as a conductive filler without surface modification. The hydrogen silicone oil in this system not only acts as the crosslinker for vinyl silicone oil but also as the anchor that locks the CCB in the crosslinked network by reacting with the hydroxyl groups on CCB's surface. The curing behavior was discussed to determine the most suitable formula to prepare the CSR. The mechanical tests revealed that CSR performed three times better in tensile strength than pure SR. The electrical conductivity was researched to choose the optimum filler content, the results exhibited that under 14 phr (phr indicates the mass ratio to vinyl silicone oil) of CCB addition, the CSR can achieve a homogenously low resistance value distribution on a large-scale area, which suggests that this CSR can be used to fabricate large-size products. A thermal stability test was carried out to assure the CSRs' performance under heat. Finally, the surface metallization effect of the samples was studied and they exhibited satisfying electrodeposition behavior of an electroplating speed of 2.40 mm/24 h. In summary, our approach is simple, cost-efficient, and is able to avoid filler migration in the SR matrix. At the same time, the uniformity of its large-area resistance distribution permits it to be used in large-scale manufacturing. To the best of our knowledge, this is the first research on the CSR used in mold manufacture as an electroplating matrix, which may broaden the use of conductive elastomers and inspire more directions of research in relative fields.

## 2. Experiment sections

### 2.1 Materials

Commercially available DY-V401-310 vinyl silicone oil (Vi–polydimethylsiloxane (Vi–PDMS), vinyl content 0.0205 mol/100 g, 350 CP viscosity at 25 °C, *M*_w_ ≈ 12 000) and DY-H212-T-0.1% hydrogen silicone oil (polyhydrogenmethylsiloxane (PHMS), 0.1 wt% hydrogen content, 120 CP viscosity at 25 °C, *M*_w_ ≈ 10 500) were purchased from Shandong Dayi Chemical Co., Ltd. Platinum catalyst PC-13B (3000 ppm) was purchased from Shanghai Neutron Star Chemical Technology Co., Ltd. AC-80 conductive carbon black (CCB) was purchased from Tianjin Tianyi Century Chemical Industry Co., Ltd. Diethoxymethylsilane (DEMS) was purchased from Aladdin Industry Co., Ltd. Toluene (AR) was used as the solvent in the swelling test.

### 2.2 Fabrication of CSR samples

**Fig. 1:**
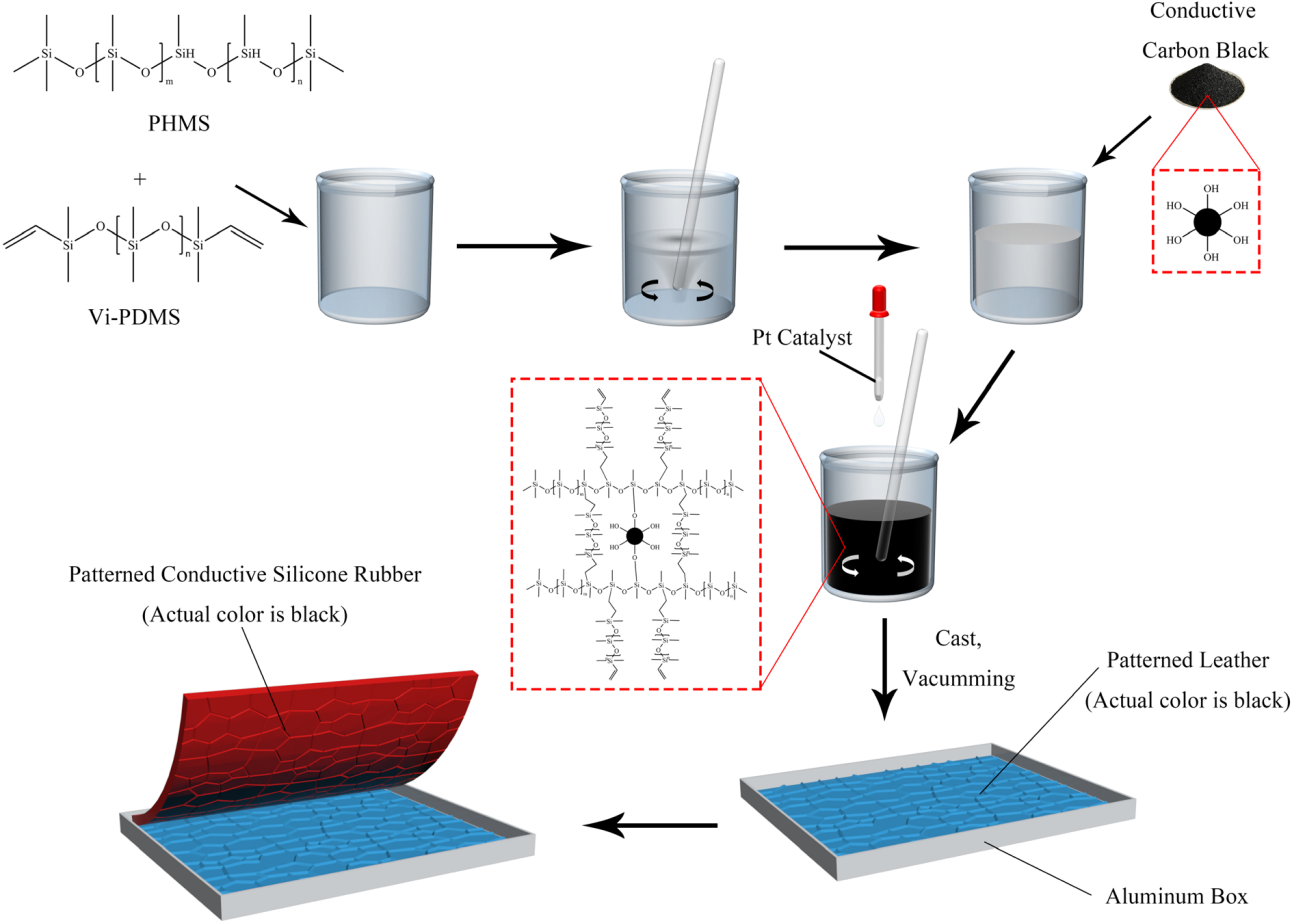
The preparation process of the CSR sample.

CSR was prepared with a certain mixing ratio of Vi–PDMS, PHMS, platinum catalyst and CCB. Vi–PDMS and PHMS were thoroughly mixed for 3 minutes by mechanical stirring. Then, CCB was added to the mixed silicone oil followed by 5 minutes of mechanical stirring. Afterward, 2 wt% platinum catalyst was added to the mixture with another 2 minutes of stirring applied. After that, the mixture was vacuumed for 5 minutes and put into a mold. Finally, the mixture was cured at 25 °C for 24 hours. After demolding, a CSR sample is obtained. The samples were named according to their formula (Table 1). The preparation process is illustrated in Fig. 1. Moreover, E-CSR-*n* means CSR-*n* after electroplating.

**Table 1:** Sample formula of SR and CSR samples

Sample	Component (g)			
	Vi–PDMS	PHMS	Platinum catalyst	CCB
SR-9	10	0.9	0.2	0
SR-10	10	1.0	0.2	0
SR-11	10	1.1	0.2	0
SR-12	10	1.2	0.2	0
SR-13	10	1.3	0.2	0
CSR-H_18_	10	1.8	0.2	1.1
CSR-H_19_	10	1.9	0.2	1.1
CSR-H_20_ (CSR-11)	10	2.0	0.2	1.1
CSR-H_21_	10	2.1	0.2	1.1
CSR-12	10	2.0	0.2	1.2
CSR-13	10	2.0	0.2	1.3
CSR-14	10	2.0	0.2	1.4

### 2.3 Equilibrium swelling method

The samples were cut into regular cubes and their volume was measured with a Vernier caliper. CSR was weighed accurately and fully swelled with toluene solution at room temperature for 3–7 days (changing solvent every other day). Samples were taken out at intervals of time and weighed after wiping the surface solvent quickly with filter paper. When the difference between the two masses' weights is not more than 0.002 g, it can be considered that the swelling equilibrium is reached.^33^

According to rubber elasticity statistics and Flory–Huggins theory,^34^ the cross-linking density of rubber can be described by eqn (1) and (2)1
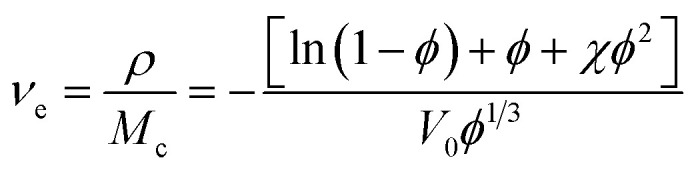
2
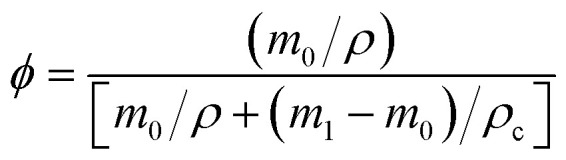
here, *ν*_e_ is the cross-linking density, (mol cm^−3^); *ρ* is the density of the original silicone rubber, (g cm^−3^); *ρ*_c_ is the density of toluene, (g cm^−3^); *M*_c_ is the average relative molecular mass between cross-linking points in silicone rubber, (g mol^−1^); the volume fraction of silicone rubber in the swelling sample is *ϕ*; *χ* is the interaction parameter between Flory–Huggins solvent and silicone rubber matrix, the selected Flory–Huggins solvent is toluene, *χ* = 0.465; *V*_0_ is the molar volume of toluene, (L mol^−1^); *m*_0_ is the initial mass of silicone rubber, (g); *m*_1_ is the quality of swelled silicone rubber, (g).

Gel volume^35^ is expressed by eqn (3), sol volume is expressed by eqn (4), and *m*_2_ is the quality of swelling silicone rubber after drying, (g).3
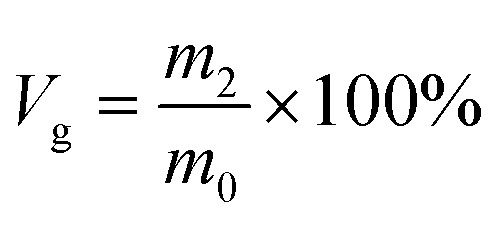
4
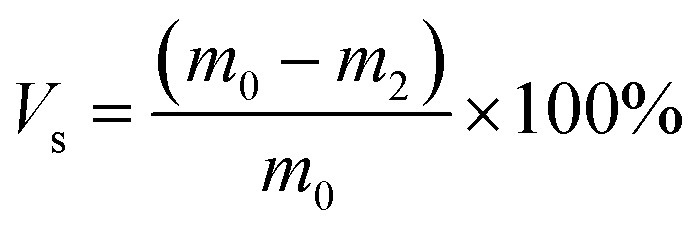


### 2.4 Electrodeposition of CSR samples

Table S1† shows the composition of the electroplating solution used for the test. The process parameters are as follows: the electroplating solution temperature is 50 °C; the electroplating liquid pH is kept between 3.8 and 4; the current density of the cathode is 2 A dm^−2^; the speed of mechanical stirring is 350 rpm; the plating time for the samples of each formula is 30 minutes and 6 hours. The back of the samples is insulated to guarantee only one side is plated when electroplating. The equipment used for electroplating is assembled as shown in Fig. S1.†

### 2.5 Characterization

The molecular weight of Vi–PDMS and PHMS was measured by a liquid chromatographer (Waters-1525, America). The microstructure of the section of the CSR and the surface of electroplated samples were examined using a scanning electron microscope (Carl Zeiss EVO18) operating at 20 kV. The SEM samples were pre-treated with a 10 nm layer of sprayed gold. The mechanical property was investigated following the standard of GB/T 528-2009/ISO 37:2005 *via* a universal testing machine (WSM-5kN). The dimension of the dumbbell sample is 115 × 6 mm. TGA was carried out under nitrogen flow (flow rate: 40 mL min^−1^) with a NETZSCH STA 449F3 (Germany) thermogravimetric analyzer. In each case, the mass of the sample was 5 to 10 mg, and the sample was heated from 30 °C to 950 °C at the rate of 10 °C min^−1^ under a nitrogen flow of 50 mL min^−1^. The resistance of CSR samples was measured by a four-probe resistance tester with the range of 0–200 kΩ on a sample size of 20 mm × 20 mm × 2 mm. Fourier transform infrared spectroscopy (ATR-FTIR, TENSOR27, Germany) was used to identify functional groups on the samples in the wave number range between 4000 cm^−1^ and 400 cm^−1^.

## 3. Results and discussion

### 3.1 Curing behavior

**Fig. 2:**
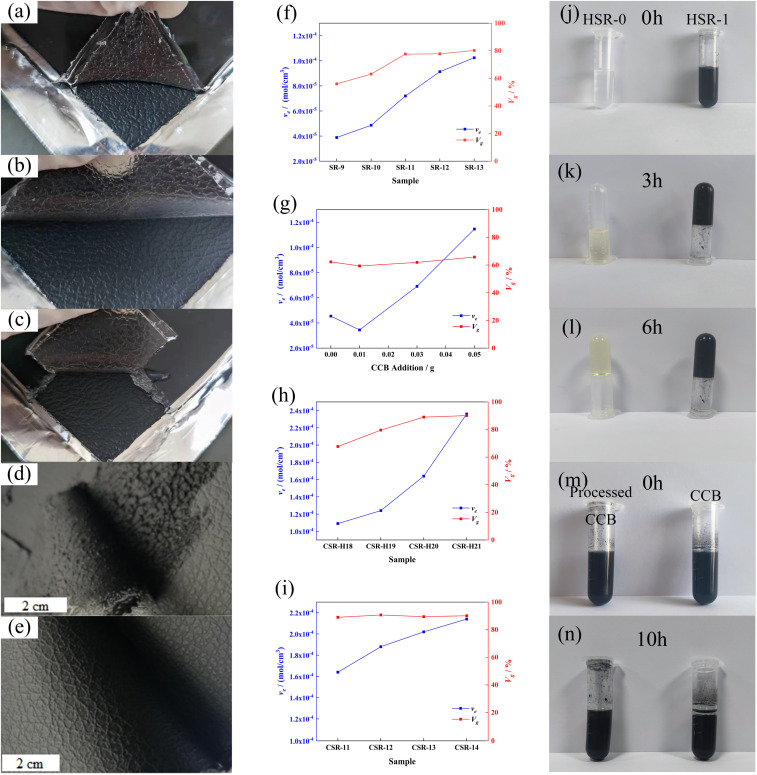
Photos of samples (a) SR-9, (b) SR-12 and (c) SR-13. Photos of (d) uncured and (e) cured CSR samples. Swelling experiment results of (f) SR-*n* samples, (g) HSR-*n* samples, (h) CSR-H_*n*_ samples and (i) CSR-*n* samples, respectively. HSR-0 and HSR-1 samples after reacting for (j) 0 h, (k) 3 h and (l) 6 h, respectively. Sedimentation experiment results after (m) 0 h and (n) 10 h, respectively.

To investigate the reason for insufficient curing of SR caused by CCB, the curing behavior of silicone rubber without CCB is discussed first. The molecular weight distribution and the FT-IR spectrum of Vi–PDMS and PHMS is shown in Fig. S2.† The digital pictures and swelling curves of different samples are shown in Fig. 2 and the data is listed in Table S2.† As shown in Fig. 2f, the gel volume (*V*_g_) and the cross-linking density (*ν*_e_) of SR samples elevate simultaneously as the addition of PHMS increases. As the photos illustrated, sample SR-9 (Fig. 2a) cannot be completely cured after 24 hours and its surface is sticky, thus it is difficult to peel it off from the patterned mold. The reason is that the SR-9 sample has the lowest *V*_g_ and *ν*_e_ in all SR samples (Fig. 2f), which suggests that the crosslinked network formation is not complete in the matrix. Sample SR-12 is fully cured and can be demolded from the mold easily without any indication of a sticky surface (Fig. 2b). The *ν*_e_ of SR-12 is over twice than of SR-9 (Fig. 2f), indicating a more sufficient formation of the crosslinked network. The *ν*_e_ of SR-13 continues to rise; however, due to the lack of reinforcing particles, SR-13 is brittle after solidifying and a fracture occurs during the demolding process (Fig. 2c). It can be concluded that the pure SR samples can fully cure once the PHMS is over 12 phr; but the lack of the reinforcing agent may cause a brittle fracture of the samples. Hence, 12 phr is the optimal addition of PHMS for pure CSR samples in this experiment.

To enable the SR with electrical conductivity, 11 phr of conductive carbon black was introduced into SR-12. However, under the same curing condition, the composite sample did not completely solidify and there was residue on the mold during demolding (Fig. 2d). In other words, the CCB interferes with the curing behavior of the composite. According to the results obtained by Jian-fang Ge and her colleagues, the silicon–hydrogen bonds can not only react with unsaturated hydrocarbons and their derivatives but also dehydrogenate with hydroxyl groups (–OH) under the presence of a catalyst to release hydrogen.^36^ In the industrial manufacturing process of CCB, abundant hydroxyl groups are introduced onto their surface. So the not curing of the sample may due to the reaction between –OH and Si–H depleting the PHMS in the system, causing an insufficient in the hydrosilylation reaction. To explore the condition of the silicon–hydrogen bonds react with the hydroxyl groups on CCB, PHMS with multiple silicon–hydrogen groups and DEMS with single silicon–hydrogen group were used for the contrastive experiments reacting with CCB (methods are described in ESI†). The self-polymerization of PHMS occurred in the presence of Pt catalyst, which can be accelerated with the existence of CCB. The black gel was formed only in 3 hours, while the transparent gel was obtained after 6 hours, as shown in Fig. 2j and k.

A set of swelling experiments was performed to demonstrate the reaction between the Si–H bond and the hydroxyl group; the formula is listed in Table S3† and the result is depicted in Fig. 2g. Since the overall addition of PHMS is the same for these samples, their *V*_g_ values are similar. However, the *ν*_e_ value in Fig. 2g continues to rise with further CCB additions. And the solvent was clear and transparent during the swelling experiments, which means there was no precipitation of CCB. The possible explanation is that the CCB reacts with the PHMS and is anchored in the cross-linking network as an extra cross-linking point.

**Scheme 1:**
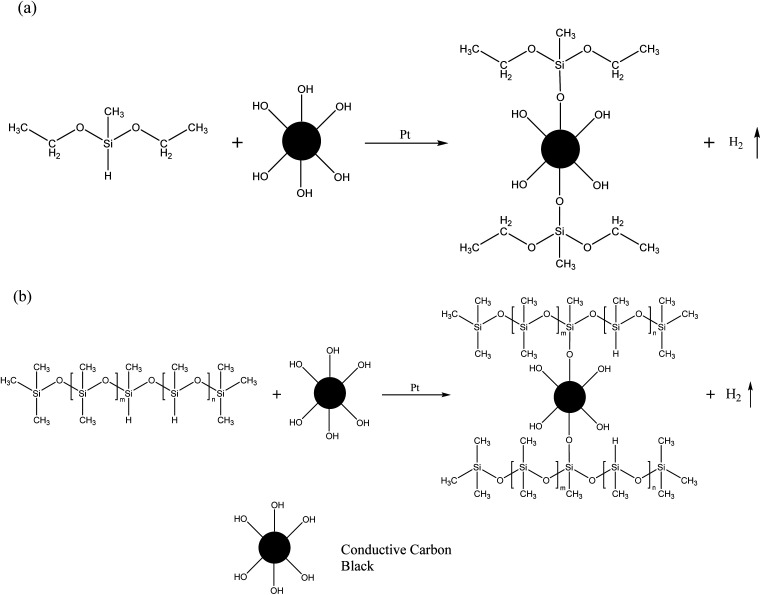
(a) The reaction between DEMS and CCB. (b) The reaction between PHMS and CCB.

To detect the changes in the functional group of CCB by spectrum, DEMS with only single Si–H groups was chosen as the carrier of the Si–H group to react with the CCB, which avoids the effect of self-polymerization of the PHMS. As Fig. S3a† depicted, hydrogen is rapidly produced immediately after the CCB and Pt catalysts are added into DEMS (Fig. S3a,† DC-1), while the production rate of hydrogen in the tube where no CCB is added is much slower (Fig. S3a,† DC-0). After 2 hours of the reaction, the DC-1 sample was still producing bubbles, whereas DC-0 stopped bubbling. The picture in Fig. S3b† shows the status of each sample when the reaction was complete. Pure DEMS is colorless and transparent (DC-N, Fig. S3b† left); DEMS with Pt catalyst is yellow and transparent (DC-0, Fig. S3b† middle); CCB has sedimented after the reaction is over and the supernatant is transparent and nearly colorless (DC-1, Fig. S3b† right). The yellow color comes from the dimer of DEMS and the color of the supernatant in the DC-1 suggests that some of the DEMS are consumed by the hydroxyl groups on the CCB. The deposited CCB was then extracted and processed for sedimentation experiments and FT-IR tests. Fig. 2m and n record the result of sedimentation experiments, pure CCB sediments in toluene while the CCB from the DC-1 sample still maintains a uniform suspension after 24 h. The result of the FT-IR test is shown in Fig. S4,† the characteristic peak at ∼3500 cm^−1^ is assigned to the hydroxyl group connected by the hydrogen bond and the intensity of it drops almost by half after the CCB reacts with DEMS. The results of these aforementioned experiments together demonstrate that the hydroxyl groups on the CCB react with the Si–H bonds, the reaction of CCB and DEMS is showed in Scheme 1a. Obviously, the CSR composite cannot be able to fully cure is that CCB consume the PHMS and cause the insufficiency of crosslinker in the system. The reaction is shown in Scheme 1b.

In order to eliminate the ill effects inflicted by the hydroxyl group on CCB, meanwhile taking advantage of the reaction to embed the CCB into the crosslinked network, additional crosslinkers were added to the reaction system. The results of the swelling experiments of the CSR-H_*n*_ samples are plotted in Fig. 2h. The CCB content in CSR-H_*n*_ samples is constant, so the impact inflicted by the reaction between Si–H and –OH is fixed. As the amount of PHMS increases, the *ν*_e_ of CSR-H_*n*_ samples escalates, indicating a more efficient formation of the cross-linking network. When the additive amount of PHMS is over 20 phr, the *V*_g_ of the CSR-H_*n*_ samples is similar, suggesting that the reaction between Vi–PDMS and PHMS is sufficient. Yet the *ν*_e_ of CSR-H_*n*_ samples still increase along with the addition of CCB, indicating that the excessive PHMS reacts with CCB and forms more crosslinked junctions in the composite. Sample CSR-H_20_ is able to meet the usage requirements for its good curing effect as well as the demolding behavior. However, the further addition of crosslinkers may cause brittle crackers during demolding. So the optimum additive amount of PHMS for CSR samples in this experiment is 20 phr.

The swelling experiments were also conducted on the CSR-*n* samples to further prove the reaction between PHMS and CCB. As shown in Fig. 2i, the CSR-*n* samples have similar gel volume of around 90%; yet the cross-linking density keeps rising, illustrating that the CCB participates in the crosslink reaction and provides additional cross-linking junctions.

### 3.2 Mechanical properties

**Fig. 3:**
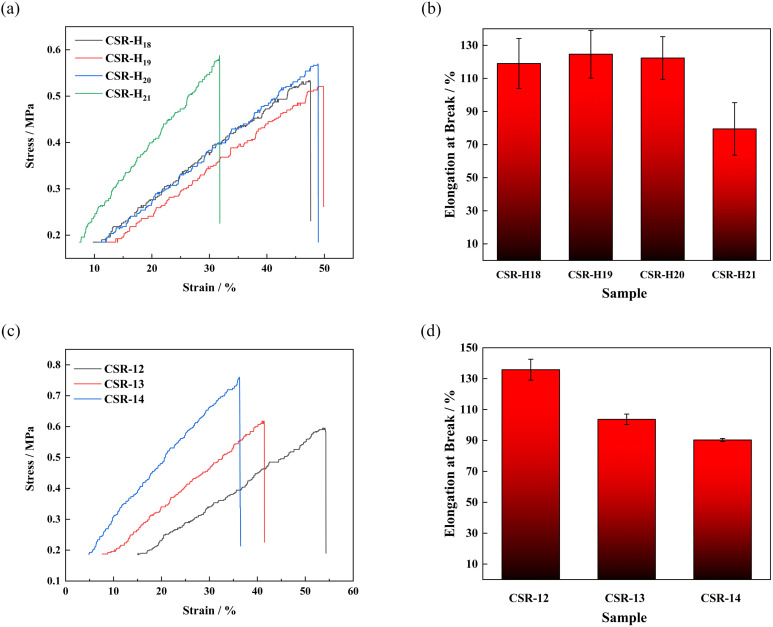
(a) Strain–stress and (b) elongation at break of CSR-H_*n*_ samples. (c) Strain–stress and (d) elongation at break of different CSR-*n* samples.

A tensile test was performed on a universal testing machine, and the results are shown in Fig. 3. The pure SR samples were too weak to be stretched that most of them shattered when the clamp squeezed, and the highest tensile strength was achieved by SR-12 (about 0.25 MPa). The introduction of CCB gains strength for the CSR samples and the most probable reason is that CCB increases the cross-linking density of CSR samples. The hydroxyl groups on the surface of the CCB interact with the Si–H bonds in the PHMS to form extra Si–O–C bonds, resulting in a denser crosslinked network in the CSR samples.

As shown in Fig. 3a and c, increasing the addition of PHMS or CCB leads to a gradual escalation of the tensile stress of the CSR-H_*n*_ or CSR-*n* samples. More crosslinkers or more CCB imply additional binding sites inside the composite, thus creating additional chemical bonds and requiring higher levels of energy to pull the sample apart. However, the denser the binding sites, the more brittle the composite. As shown in Fig. 3b, the elongation at break of the sample CSR-H_21_ drops under 80%. The explanation for this phenomenon may be that the over-dense binding sites limit the movement of the polymer chains, making sample CSR-H_21_ significantly more brittle than other CSR-H_*n*_ samples.

The CSR-*n* samples have a similar change tendency like the CSR-H_*n*_ samples, the tensile strength increases as the CCB addition gets more (Fig. 3c). The hydroxyl groups on the CCB provide additional binding sites inside the compound and enables the CSR-*n* sample to have a higher cross-linking density and tensile strength. In addition to its function as a binding site provider and electron transporter, the CCB also acts as a reinforcing particle inside the composite. The rigid CCB particles cannot be bent or twisted, and their reaction with the PHMS chains limits the movement of other polymer chains in the matrix. Therefore, the more CCB is added, the higher the tensile strength of the CSR sample. Sample CSR-14 has the highest tensile stress of 0.767 MPa, which is more than three times higher than that of the pure SR sample. At the same time, its comparative low elongation at break of 90.30% (Fig. 3d) also verifies that a higher strength comes with a weaker toughness.

### 3.3 Electrical properties

**Fig. 4:**
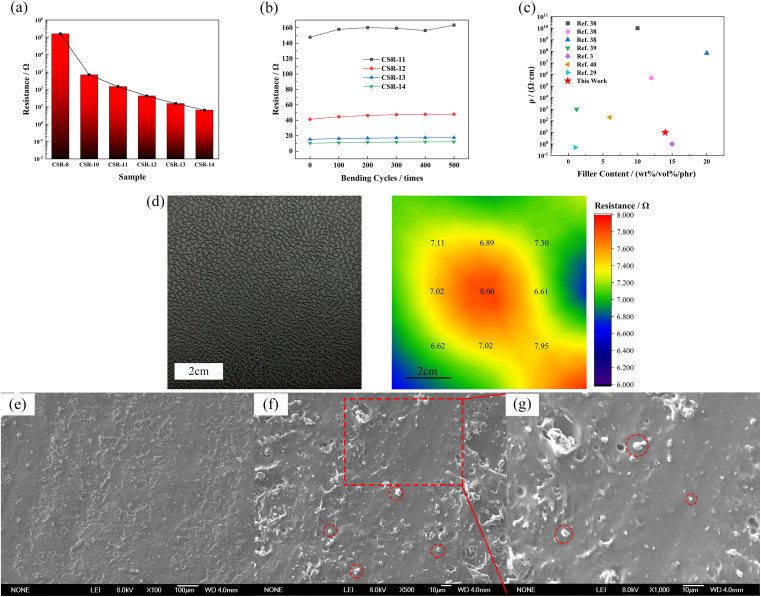
The resistance value (a) and resistance value after different bending cycles (b) of different additive amounts of CCB. (c) Resistivity comparation of previous work and this work. (d) The large size CSR-14 sample and the distribution of resistance on it (numbers are the resistance values of the sampling points). (e–g) SEM graphs of CSR-14 sample section (red circles indicate some of the CCB particles), (e) 100×, (f) 500×, (g)1000×.

The resistance values of CSR samples are shown in Fig. 4a. The sample name is denoted as CSR-*n*, and *n* is the amount of CCB added. It can be seen from Fig. 4a that with the additive amount of CCB rising, the resistance of CSR samples went through a progress from around 2 × 10^5^ Ω to a low value of 10.20 Ω. This can be explained by the percolation threshold theory.^37^ The sample CSR-8 is nearly insulating, suggesting its filler content is below the percolation threshold. The resistance value rapidly dropped by three orders of magnitude (CSR-8 to CSR-11) as more CCB was added into the substrate, which demonstrates that the percolation threshold was reached and the composite was able to conduct electricity. This is because the increase of CCB shortens the distance between conductive particles so it is easier for electricity to run through the composite, as shown in Fig. S5a and b.† However, the decrease rate of resistance value slowed down after CCB reaches 11 phr, which is in accordance with the third stage in the percolation curve. Fig. S5b and c† also exhibit similar distances between CCB particles.

Fig. 4b demonstrates that the resistance values of CSR samples fluctuate within a narrow range during five hundred bending cycles, indicating that the CSR samples have excellent stabilities. This phenomenon occurs because the CCB is locked in the crosslinked network *via* the reaction between the hydroxyl groups it carries and the Si–H bonds in the PHMS so that the filler particles do not migrate through the composite samples under external forces. The resistivity of CSR-14 is 9.80 Ω cm, which is comparably low in conductive elastomers^3,38–41^ (Fig. 4c and Table S4†), indicating a fairly continuous and perfect conductive network presence inside the CSR composite. In the meantime, the distribution of large-area resistance is illustrated in Fig. 4d, and from the cloud image (color bar ranges from 6.00 to 8.00 Ω) it is shown that the resistance is uniform throughout the whole area (7.17 ± 0.48 Ω). All these can be explained by the SEM graphs of section area of sample CSR-14 (Fig. 4e–g). In the graphs, the bright dots are evenly dispersed across the whole region and the distances between them are close so that the resistance value of sample CSR-14 is low and uniform over a large area. So the best electrical properties are achieved in this experiment with the CCB addition of 14 phr.

Apart from the uniform distribution of filler in the composite, the reason for the low resistivity also lies in the fact that the pristine CCB used in the experiment is highly electrical-conductive and was not being surface-modified before mixed into the elastomer matrix. According to the theory proposed by Simmons,^42^ a model that describes the composite resistance can be formed,^43,44^ as shown in eqn (5) and (6).5
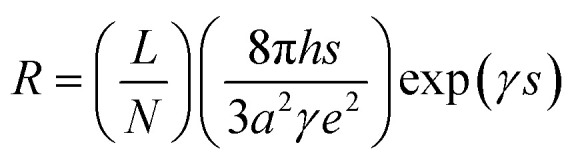
6
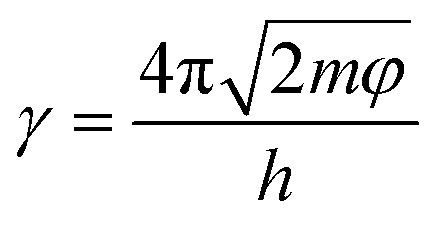
where *L* represents the total number of conductive particles forming a single conductive pathway; *N* is the number of conductive pathways inside the polymer matrix; *h* is the plank constant; *s* for the thickness of the insulating film; *m* and *e* are the electron mass and charge, respectively; *a*^2^ is the effective cross-section area and *φ* represents the height of the potential energy barrier between the two neighboring particles. It is the insulating barrier thickness that affects the electron tunneling probability so that all tunneling can be considered to occur within a tiny surface region. Therefore, *s* in eqn (5) can be thought of the minimum distance of the surface of two neighboring conductive particles. Surface modification of particle often increases its diameter, and most regents that are used for surface modification are either non-conductive or semi-conductive. Under the situation of particles equally distributed inside the matrix, the distance between the surface of two adjacent particles (no matter surface-modified or not) stays the same. However, the surface-modified CCB often gets encapsulated inside the modifier, meaning that the distance between two adjacent conductive particles is enlarged. The enlargement of distance implies an increase in the value of *s* and causes a higher resistance value *R*. To get over this problem, substances with elevated conductivity (such as Ag NP) are often grafted on the covering layer.^28^ However, grafting introduces a new complication of poor interaction with the polymer matrix. Thus, the use of pristine conductive particles is sometimes a better option.

### 3.4 Thermal properties

**Fig. 5:**
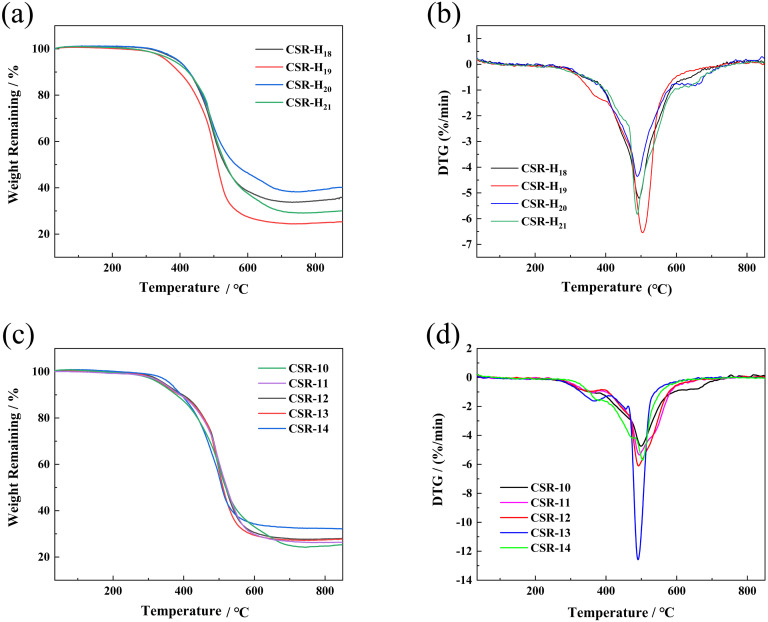
TGA curves of (a) CSR with different PHMS content and (c) CSR with different CCB content. DTG curves of (b) CSR with different PHMS content and (d) CSR with different CCB content.

TGA and DTG analyses were performed to explore the thermal stability of the CSR composite and the curves are shown in Fig. 5. The thermal decomposition of the CSR is divided into two stages. The first stage of 100–300 °C corresponds to the decomposition of the alkyl bond, which is the cleavage of the Si–C bond and produces alkane derivatives. The second decomposition stage of 300–600 °C is caused by the fracture and rearrangement of the Si–O–Si main chain in the cross-linked network. The unshared electron pairs of the oxygen atoms of the Si–O–Si backbone at high temperature coordinate with the 3d empty orbitals of the neighboring silicon atoms to produce a small molecule of cyclic siloxane, hexamethylcyclotrisiloxane D_3_.^45^ As can be seen from Fig. 5a and b, the rate and amount of CSR decomposition decreases as the crosslinker increases, indicating a negative relationship between the cross-linking density and the CSR decomposition process. This is because the increase in cross-linking density indicates a more sufficient reaction between the Vi–PDMS and the PHMS, so the amount of gel is increased and a denser network is formed. At the same time, the likelihood of degradation in CSR decreases. As for the CSR with different additive amounts of CCB (Fig. 5c), the residue at 800 °C of CSR-14 (32.39%) is the highest among all the CSR samples. In the meantime, CSR-14 has the highest temperature (503.93 °C) at the maximum decomposition rate, meaning the CSR-14 sample is the most stable under high temperatures (Fig. 5d). This phenomenon can be explained by the interaction between the CCB particles and the cross-linked rubber structures. On the one hand, the CCB particles limit the motion of the silicone rubber molecular chain and may improve the thermal stability of the CSR. On the other hand, the addition of CCB also increases the crosslinking density of the composite and levels up its heat-resistant ability.

### 3.5 Surface metallization

Finally, a surface metallization experiment was carried out to see the overall performance of the CSR samples. The electroplating results are listed in Table 2, and images of the electrodeposition results are presented in Fig. S6, S7,† and 6. The surface metallized CSR sample is named as E-CSR-*n*, and *n* is the additive amount of CCB in CSR composites (10–14 phr). During the electrodeposition process, the higher the current density is, the faster the electrodeposition proceeds. However, if the current density is too high, cathode member sintering and pulverization of electrodepositing layer can occur. When a professional Hull-cell copper plate was used as the cathode to test the electrodeposited nickel at a current density of 2 A dm^−2^, the electrodepositing rate and the surface metallization effect were the best. According to Table 2, E-CSR-10 requires a lengthy time of 104 minutes to reach the target current density of 2 A dm^−2^. When the additive amount of CCB is increased to 12 phr or more, the sample is able to achieve the optimum current density at the beginning of electrodeposition (Fig. S6a–c†). The stable voltage of the professional Hull-cell copper is between 1.1 V and 1.3 V at the optimum current density with the application of an electrolyte solution system in the surface metallization experiment. Samples E-CSR-12, E-CSR-13 and E-CSR-14 also have stable voltages in the range 1.1–1.3 V. It indicates that these three samples met the basic requirements of effective electrodeposition, that is, the CSR can achieve the electrodeposition effect of the professional Hull-cell copper sheet when the resistance value is less than 91.3 Ω. For the same current density, the electrodeposition rate depends on the cathode resistance value, and the smaller the value of the cathode resistance, the faster the electrodeposition. Sample E-CSR-14 has the highest electrodeposition rate of 2.40 mm/24 h.

**Table 2:** The electroplating result of E-CSR

Sample	Initial current density (A dm^−2^)	Initial voltage (V)	Time (min)-2 A dm^−2^	Final voltage (V)-2 A dm^−2^	Plating speed (mm/24 h)
E-CSR-10	0.39	32.0	104	2.9	0.25
E-CSR-11	1.55	32.0	14	1.8	0.27
E-CSR-12	2	22.5	0	1.3	0.83
E-CSR-13	2	11.5	0	1.3	1.32
E-CSR-14	2	4.8	0	1.2	2.40

**Fig. 6:**
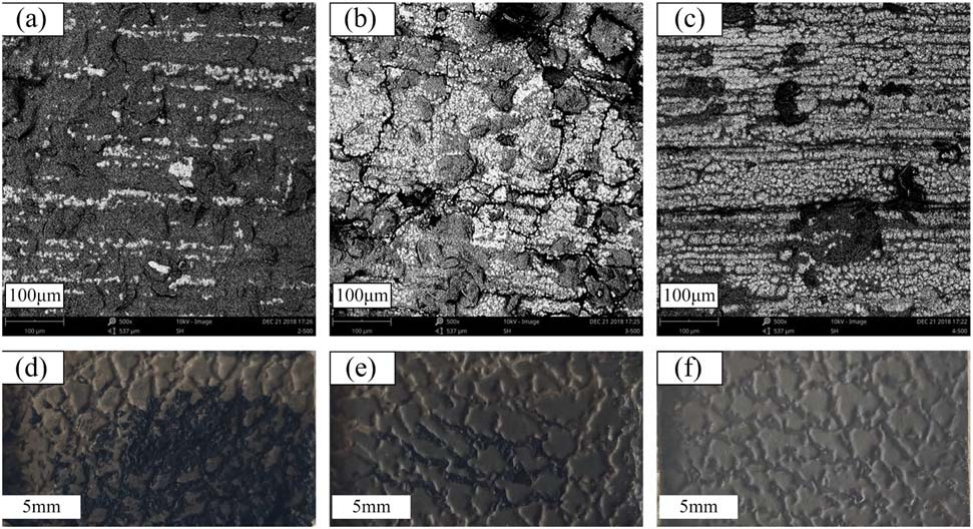
SEM graphs of CSR after electrodeposition for 30 minutes ((a) E-CSR-12; (b) E-CSR-13; (c) E-CSR-14) and photos of nickel layer after electrodeposited for 6 hours: (d) E-CSR-12; (e) E-CSR-13; (f) E-CSR-14.

As can be seen from Fig. S7† and 6a–c, samples E-CSR-12, E-CSR-13 and E-CSR-14 are able to observe significant traces of nickel. Sample E-CSR-14 has the best surface metallization effect, and nickel is able to form a continuous phase above it. Sample E-CSR-13 is also able to observe the growth state of nickel but in a less continuous form. Further experiments were carried out to see the effect of pattern duplication during electrodeposition, and samples E-CSR-12, E-CSR-13 and E-CSR-14 were electrodeposited for 6 hours to form relatively firm nickel layers. The results are shown in Fig. 6d–f. As demonstrated in the figures, all three samples can form continuous and thick layers with precisely replicated patterns. This result can be attributed to the fact that the position of the CCB particle inside the polymer matrix is fixed by the reaction between the hydroxyl groups on the surface of the CCB and the silicon–hydrogen bonds in the substrate. The anchoring of the CCB particle prevents it from migrating due to the applied voltage, so the surface morphology of the CSR remains unchanged and the pattern on the surface of the CSR can be accurately reproduced during the electroplating process.

However, samples E-CSR-12 and E-CSR-13 exhibit residuals of CSR on the layer, and the lower the filler content is, the more CSR residuals exist. The reason for this phenomenon may lie in the fact that E-CSR-12 and E-CSR-13 had higher initial voltage than that of E-CSR-14 (Table 2). Some areas of the CSR sample got burnt by the high voltage and then an SR degradation took place, followed by a CSR adherence onto the layer, making it difficult to demold.

Thus, combining all the above factors, the optimal formula for obtaining the desired CSR in this experiment is 100 phr Vi–PDMS, 20 phr PHMS, 2 phr Pt catalyst and 14 phr CCB.

## 4. Conclusions

In this work, a CSR with CCB as filler was prepared by the co-curing method. The curing behavior was first investigated, where it was found that the pure SR samples can reach the best demolding performance under the PHMS additive amount of 12 phr. The addition of CCB interferes with the curing performance of the CSR samples, and the reason lies in the fact that the hydroxyl groups on CCB react with Si–H bonds in PHMS with the existence of Pt catalyst, causing the cross-linking reaction to being insufficient. So the appropriate PHMS addition for CSR is 20 phr. The resistance value of CSR decreases as the additive CCB increases. When the CCB added is 14 phr, the resistance value is as low as 10.20 Ω and did not change much after repeated bending of 500 times, which demonstrates good repeatability and durability. Thermal tests revealed a positive relationship between thermal stability and the additive amount of crosslinker or CCB. Finally, the CSR can achieve the same electrodeposition effect as the professional-grade Hull-cell copper sheet once the resistance value is under 91.30 Ω. The maximum achievable electrodeposition rate with the CSR sample is 2.40 mm/24 h, and CSR achieves the best electrodeposition effect with a CCB addition of 14 phr. A simple fabrication process and low large-area-homogeneous resistivity combined with good stability make this silicone rubber composite suitable for precision mold manufacture, electronic devices, and other related fields.

## Conflicts of interest

The authors declare that they have no conflicts of interest.

## Supplementary Material

RA-012-D2RA06510J-s001
