# Supplementary material for: Preparation of carbon black/silicone rubber composites with large-area-homogeneous-low electrical-resistance used as electroplating matrix
Source: RSC Adv. 2022 Nov 11;12(50):32448–58. doi: 10.1039/d2ra06510j (PMC9650633; doi:10.1039/d2ra06510j)
Supplement: RA-012-D2RA06510J-s001 [file RA-012-D2RA06510J-s001.pdf]

Electronic Supplementary Material (ESI) for RSC Advances.
This journal is © The Royal Society of Chemistry 2022

Preparation of Carbon Black/Silicone Rubber Composites with Large-Area-Homogeneous-Low Electrical-Resistance used as Electroplating Matrix

Yanli Dou[1], Shixiang Sun[1], Shanshan Lu[2], Weiguo Yao[1], Dongbo Guan[1,*]
[1]The ministry of education key laboratory of automotive material; College of Material Science and Engineering, Jilin University, Changchun PR China. 130025
[2] China FAW Group Corporation R and D Center: First Automobile Works Group Corporation Research and Development Center, Changchun PR China. 130000
Correspondence: guandb@jlu.edu.cn

1. Contrastive Experiments

1.1 Materials

Commercially available DY-H212 hydrogen silicone oil (polyhydrogenmethylsiloxane (PHMS), 0.1wt% hydrogen content, 120 CP viscosity at 25°C, Mw≈10500) was purchased from Shandong Dayi Chemical Co., Ltd. Platinum catalyst PC-13B (3000 ppm) was purchased from Shanghai Neutron Star Chemical Technology Co., Ltd. AC-80 Conductive carbon black (CCB) was purchased from Tianjin Tianyi Century Chemica Industry Co., Ltd. Diethoxymethylsilane (DEMS) was purchased from Aladdin Industry Co., Ltd. Toluene (AR) was used as solvent in the tests.

1.2 Preparation of the samples

Samples for the experiment were prepared according to the formula listed in Table S2. For HSR-n samples, PHMS was first mixed with CCB for 1 min, then Pt catalyst was added to the blend and stirred to start the crosslinking process. For DC-n samples, DEMS was added into the tube of weighted CCB and stirred for 1min to ensure the formation of a primary full mixture, then Pt catalyst was added into the system and stirred for another 1min. Photos were taken by the system camera of a cell phone.

1.3 Swelling Experiment and Sedimentation Experiment

Swelling experiment was conducted as described in the manuscript.

To carry out the sedimentation experiment, DC-n samples were taken out from the DEMS then washed with ethanol and suction-filtrated for five times. Next, the samples were dried in a vacuum oven at 60 °C for 6h. Finally, the powder was added to toluene and super-sonicated for 30 min to achieve a uniform dispersion for the sedimentation experiment. Photos were taken by the system camera of a cell phone at regular intervals.

2. Supplementary Results

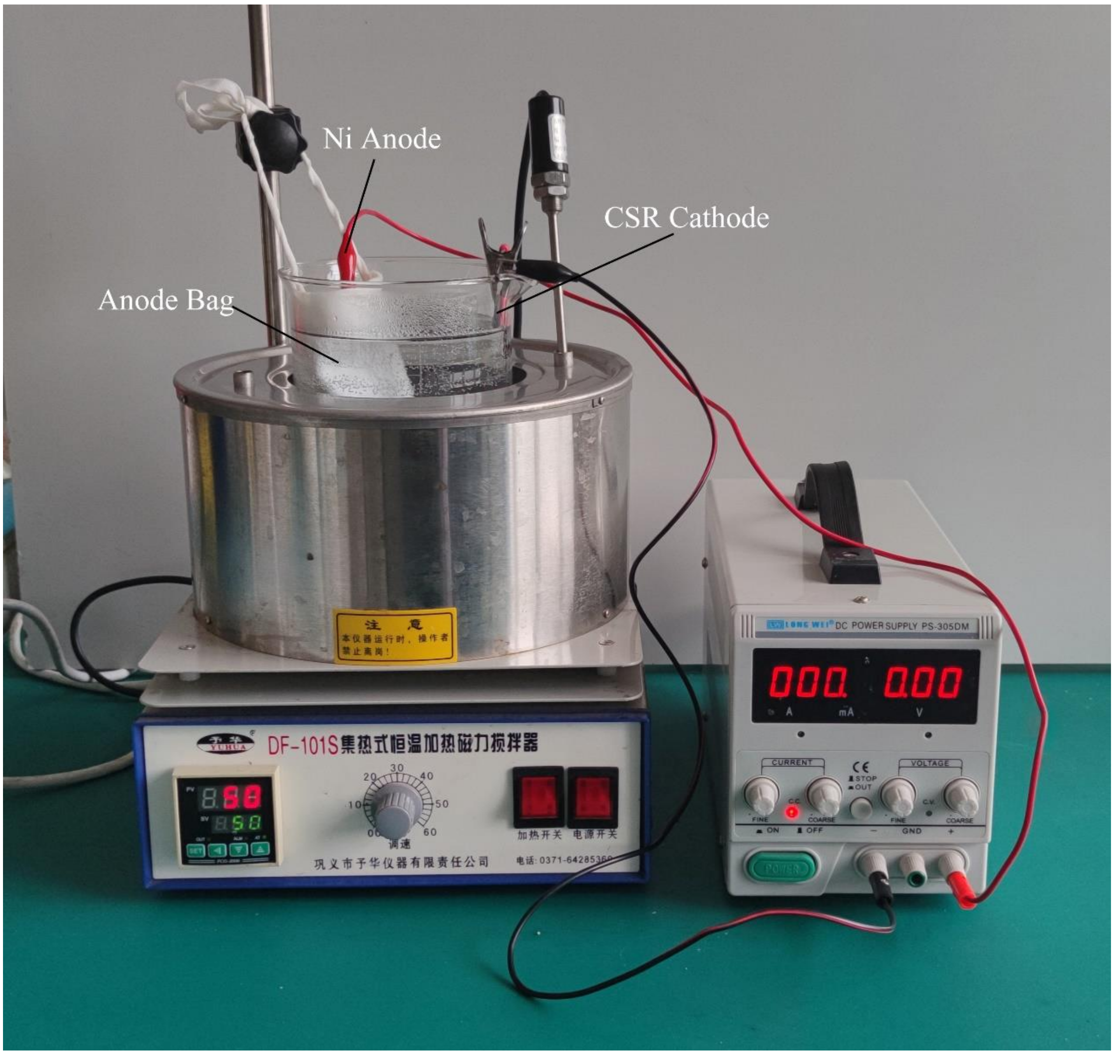

Fig. S1 An illustration of electroplating equipment used in the experiment (In order to clearly show the composition of the device, the liquid in the beaker was changed from electroplating solution to deionized water).

Table S1. Composition of electroplating solution.

Component	Concentration （g/L）
$Ni(SO_3NH_2)_2 \cdot 4H_2O$	420
$NiCl_2 \cdot 7H_2O$	5
H_3BO_3	36
$CH_3(CH_2)_{11}OSO_3Na$	0.3

(a)

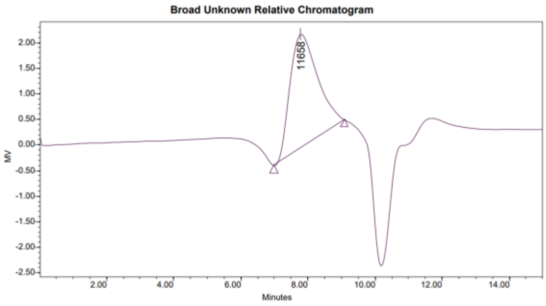

Broad Unknown Relative Peak Table

	Distribution Name	Mn (Daltons)	Mw (Daltons)	MP (Daltons)	Mz (Daltons)	Mz+1 (Daltons)	Polydispersity	Mz/Mw	Mz+1/Mw
1		6181	11899	11658	18766	25034	1.925261	1.577065	2.103838

(b)

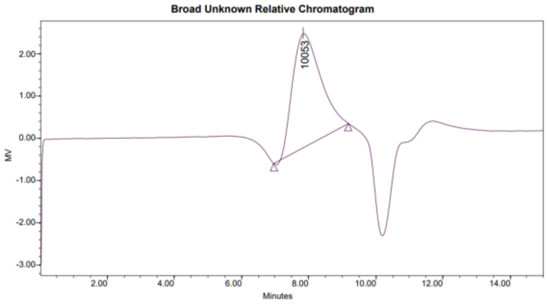

Broad Unknown Relative Peak Table

	Distribution Name	Mn (Daltons)	Mw (Daltons)	MP (Daltons)	Mz (Daltons)	Mz+1 (Daltons)	Polydispersity	Mz/Mw	Mz+1/Mw
1		5322	10421	10053	16625	22309	1.958205	1.595389	2.140797

(c)

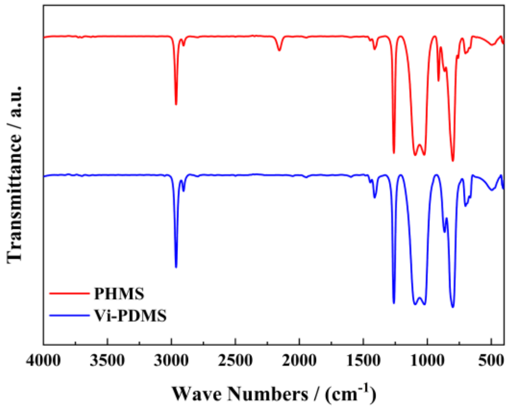

Fig. S2 Liquid Chromatogram of (a) Vi-PDMS and (b) PHMS; (c) FT-IR spectrum of Vi-PDMS and PHMS.

Table S2 Sample Crosslinking performance parameters.

Sample	V_g	v_e / (mol·cm^{-3})
SR-9	56.02%	3.885×10^{-5}
SR-10	63.10%	4.861×10^{-5}
SR-11	77.59%	7.196×10^{-5}
SR-12	77.79%	9.131×10^{-5}
SR-13	80.21%	1.024×10^{-4}
CSR-H_{18}	67.70%	1.09×10^{-4}
CSR-H_{19}	79.60%	1.24×10^{-4}
CSR-H_{20} (CSR-11)	89.00%	1.64×10^{-4}
CSR-H_{21}	90.10%	2.36×10^{-4}
CSR-12	90.70%	1.88×10^{-4}
CSR-13	89.46%	2.04×10^{-4}
CSR-14	90.13%	2.14×10^{-4}

Table S3 Sample Formula for Swelling and Sedimentation Experiments.

Sample	Components (By Mass / g)			
	PHMS	DEMS	CCB	Pt catalyst
HSR-0	1	0	0	0.1
HSR-1	1	0	0.01	0.1
HSR-2	1	0	0.03	0.1
HSR-3	1	0	0.05	0.1
DC-N	0	1	0	0
DC-0	0	1	0	0.1
DC-1	0	1	0.01	0.1

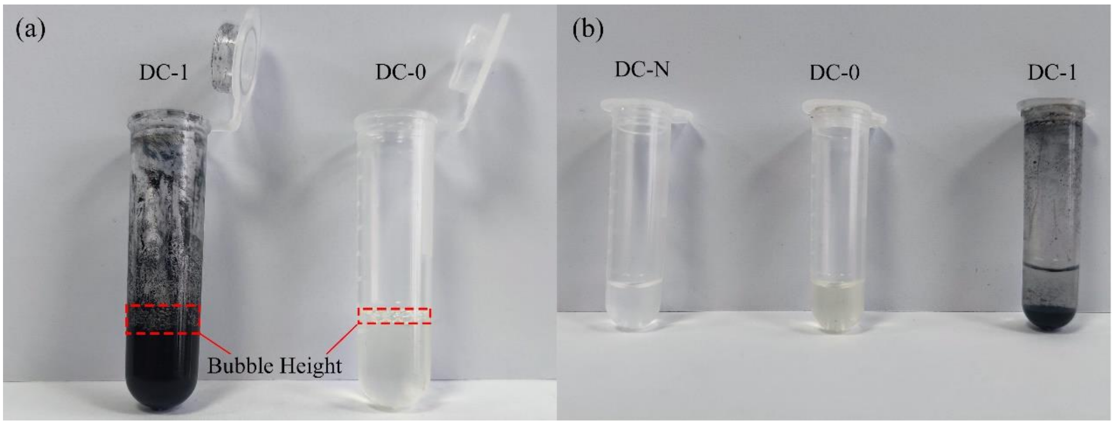

Fig. S3 (a) Bubble generation speed of DC-0 (right) and DC-1 (left) samples (bubble height indicates the bubble generation speed). (b) Status of DC-N (left), DC-0 (middle), DC-1 (right) samples when the reaction is complete.

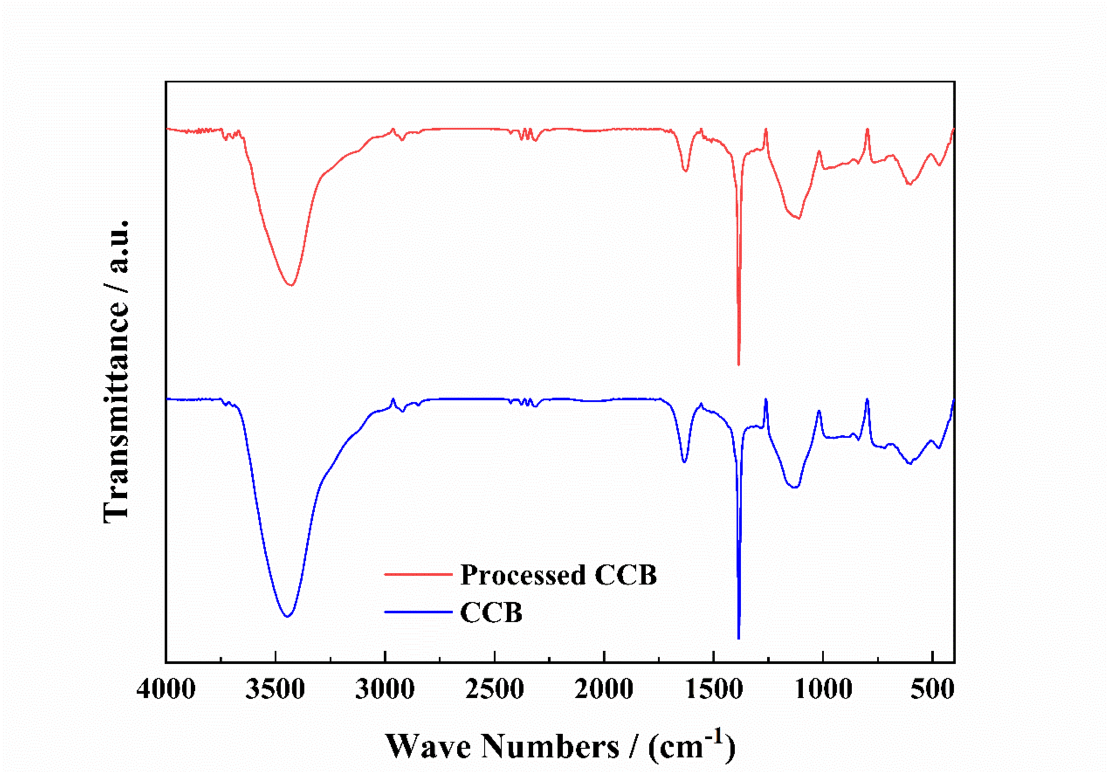

Fig. S4 FT-IR spectrum of processed CCB (CCB that was taken out of DC-X sample then washed and dried) and raw CCB.

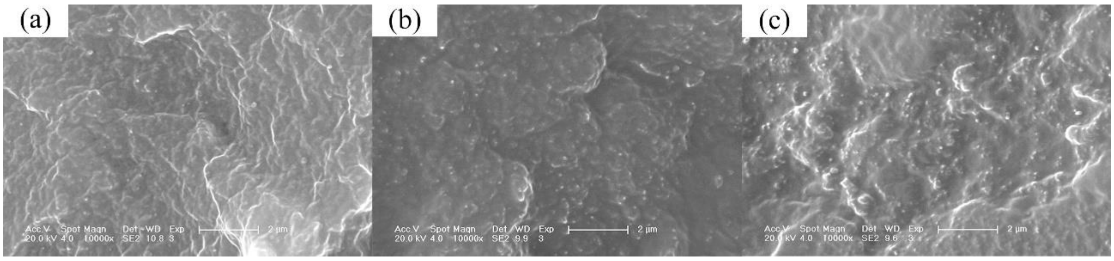

Fig. S5 Section morphology of CSR with different CCB contents (a. CSR-8; b. CSR-11; c. CSR-13).

Table S4 The comparison of conductive elastomers using carbon fillers.

Substrate	Filler type	Resistivity (Ω·cm)	Filler content	Ref.
PAN	CCB	~1010	10 wt%	[1]
PAN	BZCB	$\sim 5 \times 10^{5}$	12 wt%	[1]
PAN	Ag-ABCB	$\sim 7 \times 10^{7}$	20 wt%	[1]
TPU	CB	~103	1.2 vol%	[2]
SR	CCB-PMPS/CNT	~1	15 wt%	[3]
SR	CNT/Graphene	~200	6 wt%	[4]
SR	CNT/Graphene	0.5	1 wt%	[5]
SR	CCB	9.80	14 phr	This work

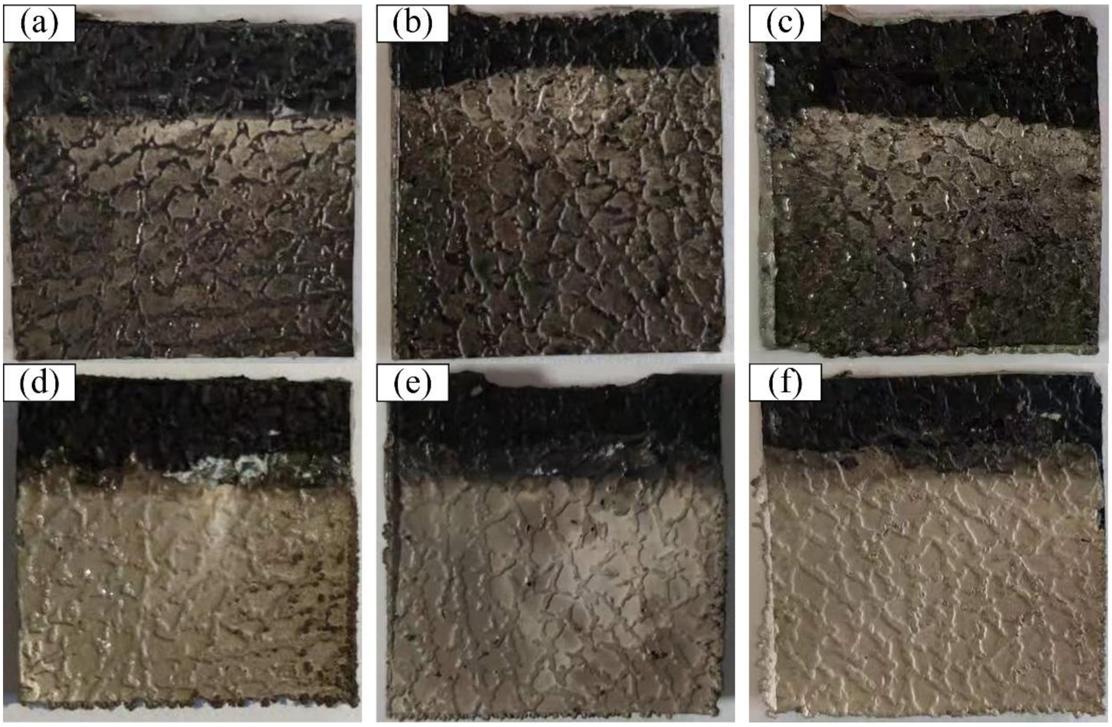

Fig. S6 Photos of CSR after electrodeposition for 0 minutes (a. E-CSR-12;b. E-CSR-13 ;c. E-CSR-14) and photos of CSR after electrodeposition for 4h (d. E-CSR-12;e. E-CSR-13 ;f. E-CSR-14).

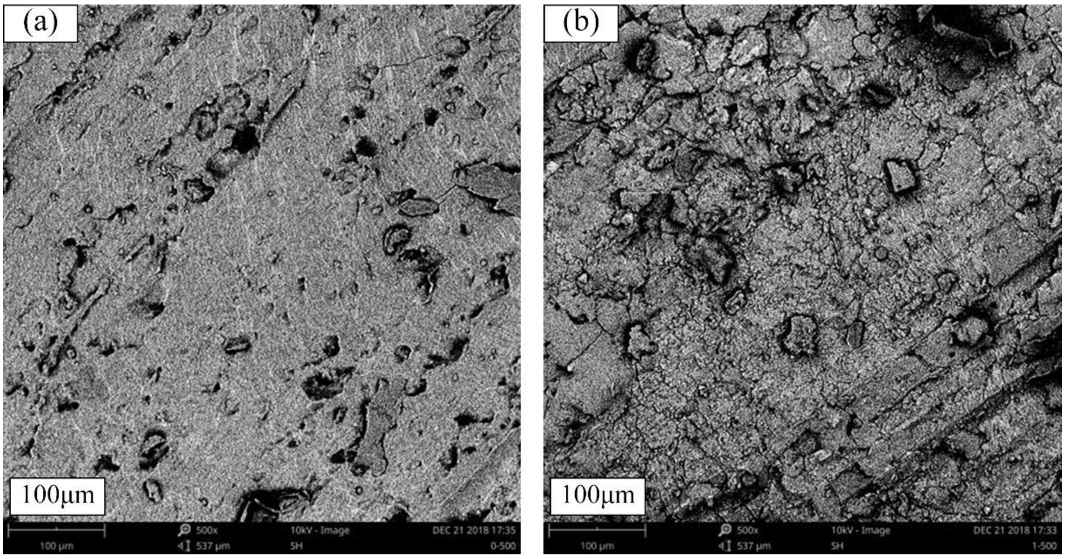

Fig. S7 SEM graphs of CSR after electrodeposition for 30 minutes (a. E-CSR-10; b. E-CSR-11)

1. Ahn D, Choi HJ, Kim HD, Yeo SY (2020) Properties of Conductive Polyacrylonitrile Fibers Prepared by Using Benzoxazine Modified Carbon Black. Polymers (Basel) 12 (1). doi:10.3390/polym12010179

2. Zhai Y, Yu Y, Zhou K, Yun Z, Huang W, Liu H, Xia Q, Dai K, Zheng G, Liu C, Shen C (2020) Flexible and wearable carbon black/thermoplastic polyurethane foam with a pinnate-veined aligned porous structure for multifunctional piezoresistive sensors. Chemical Engineering Journal 382. doi:10.1016/j.cej.2019.122985

3. Song P, Song J, Zhang Y (2020) Stretchable conductor based on carbon nanotube/carbon black silicone rubber nanocomposites with highly mechanical, electrical properties and strain sensitivity. Composites Part B: Engineering 191. doi:10.1016/j.compositesb.2020.107979

4. Yang H, Yao X, Yuan L, Gong L, Liu Y (2019) Strain-sensitive electrical conductivity of carbon nanotube-graphene-filled rubber composites under cyclic loading. Nanoscale 11 (2):578-586. doi:10.1039/c8nr07737a

5. Oh JY, Jun GH, Jin S, Ryu HJ, Hong SH (2016) Enhanced Electrical Networks of Stretchable Conductors with Small Fraction of Carbon Nanotube/Graphene Hybrid Fillers. ACS Appl Mater Interfaces 8 (5):3319-3325. doi:10.1021/acsami.5b11205
